# High Potencies in Acute as Well as Chronic Diseases

**Published:** 1882-06

**Authors:** A. J. Brewster

**Affiliations:** Syracuse, N. Y.


### THE HIGH POTENCIES IN ACUTE AS WELL AS CHRONIC DISEASES.

A. J. Brewster, M. D., Syracuse, N. Y.

(*Read before the New York Central Homœopathic Society, at their Quarterly Meeting, held March* 16*th*, 1882.)

It is no longer a matter of faith with me that the high potencies act promptly and curatively when properly selected and administered. The fullness of a long experience has established the fact that higher and the highest potencies not only act, but act powerfully and curatively, upon violent forms of disease, both acute and chronic. What we learn from patient and persistent experiment and observation, becomes to us knowledge. We walk not by faith but by sight in *this* matter. And not only does the physician *see*, but the patient sees, the effect of the remedies, and rejoices in the relief it brings, and it becomes knowledge to them also. To be a little more definite, we will relate some clinical experience.

At the close of a sultry day in July last, we stood at the bedside of a Mr. T., aged about thirty years, a farmer, of good constitution and of good general health. He had, for some days, felt darting pains in the left testicle and cord, going up, like electric shocks, into the left side of the abdomen, and around to the left side of the back and kidney, coming and going suddenly, with intervals of comparative relief from pain. On this day, the pains getting more violent and continuous, the writer was called in haste. We found him in the following condition, *pathologically:* The left testicle enlarged and the spermatic cord badly swollen; the parts red and very sensitive, accompanied by fever. This condition, 'tis true, suggested a class of remedies, but could give us nothing definite as to *the* remedy in *this* case; but the accompanying symptoms guided us unmistakably to the simillimum. In Hering's condensed *Materia Medica*, we find this group of symptoms, which covered the symptoms of the case so perfectly, that we were confident of satisfactory results: Violent stitches in the testicle, which is drawn upward; tearing upwards in the left spermatic cord in the evening in bed; violent pressing, urging toward the genitals, as though everything would fall out; soreness of the last dorsal and first lumbar vertibræ; backache as if broken, with cramp-like sensation in the left lumbar region, red tongue, flushed face and glistening eyes, led us back to Belladonna for relief, and it did not disappoint us.

Belladonna$^{14c}$ was given, with prompt relief; but *it did not cure.* The above symptoms, with some others, which had been more or less troublesome for some months, continued, in a less degree, to be troublesome and to annoy the patient.

In reviewing the case, additional symptoms were discovered, viz.: a want of power to evacuate the bowels, pressing in the rectum toward the anus. Voluptuous tickling in the lower parts of the rectum and anus. Spasmodic constriction of the sphincter ani, mucous membrane of the anus seems swollen and pressed out. Violent itching, at the same time constrictive sensation in the anus. Frequent desire to urinate, with small quantity. Vesical region very sensitive to pressure and to jar. All of these symptoms were covered by Bell. and Swan's MM potency; a powder night and morning, dry, for four days, removed the whole group completely.

We are often asked: Can the high potencies produce aggravations?

Several days after receiving the Bell. high, our patient reported at the office that he had not felt so well and been so free from pain in months. Bowels natural, urine free and painless, swelling and pains in the parts all gone, only a little weakness of the back remaining. Indeed, he felt quite like himself again, but as he lived six miles from town he asked for some more of those little powders, for he feared the return of the dreadful pains which made him feel as though he was called to go hence, and was not quite ready. He received some with instructions not to take them unless the pain returned and then only two per day. Some days after, not feeling quite as well, he commenced taking them and *reasoning*, as do *many*, that if so little medicine could make him feel so *good*, more would make him feel a good deal better, so he continued to take them four times per day for four or five days. The result was after taking the powders for the few days he reported that all his old troubles had returned with redoubled violence. That this was a clear case of medicinal aggravation was evident to me by the whole group being removed by a single dose of the proper antidote, Hyoscyamus,$^{1m}$ and a good supply of Sac. Lac. powders. A few days after he returned with a smiling face, rejoicing that he felt himself again.

The second case is one of diphtheria. During the spring of '81, was called to see a young lady suffering from a severe attack of this disease. After some general rigors and the fever that accompanies, the disease located on the left side, the parotid was swollen and very sensitive to the touch; the tonsil was also swollen, of a purplish-red color and extremely sore; deglutition very difficult. The left side of the neck swollen, very sore and dark purple or bluish in color. Violent pains darting up the left side of the neck into the head, drawing the head around to the left side and holding it there. Great prostration and the characteristic deposit on the left tonsil with aggravation of all of the symptoms during sleep. All these symptoms were covered by Lachesis and this remedy, in 2000th potency, was prescribed and prompt relief was the result. The case went on for the space of two days without improvement but rather getting worse. The symptoms not having changed no other remedy could be given. After looking the case over carefully we came to the conclusion that the severity of the symptoms called for greater dynamic force, but how could we look for greater curative power but in a higher potency? So, to satisfy ourselves on this point and to, if possible, cure the patient, which was far from being an accomplished fact, Lachesis,$^{cm}$ was administered, one dose dry on the tongue at night, and awaited the result. Morning found the case much improved, but in the evening some return of the symptoms made it necessary to repeat the dose and we continued it morning and evening for a few days, at which time we were able to pronounce the patient convalescent. Disease force had been met by dynamic force and the former had yielded to the latter.

